# Granulocyte colony-stimulating factor exacerbates hematopoietic stem cell injury after irradiation

**DOI:** 10.1186/s13578-015-0057-3

**Published:** 2015-11-25

**Authors:** Chengcheng Li, Lu Lu, Junling Zhang, Song Huang, Yonghua Xing, Mingfeng Zhao, Daohong Zhou, Deguan Li, Aimin Meng

**Affiliations:** Institute of Laboratory Animal Science, Peking Union Medical College and Chinese Academy of Medical Science, Beijing, China; Institute of Radiation Medicine, Peking Union Medical College and Chinese Academy of Medical Science, Tianjin, China; Tianjin Key Laboratory of Radiation Medicine and Molecular Nuclear Medicine, Tianjin, China; The First Central Clinical College of Tianjin Medical University, Tianjin First Central Hospital, Tianjin, China; Pharmaceutical Sciences and Winthrop P. Rockefeller Cancer Institute, University of Arkansas for Medical Sciences, Little Rock, USA

**Keywords:** G-CSF, HSC, Ionizing radiation

## Abstract

**Background:**

Exposure to a moderate to high dose of ionizing radiation (IR) not only causes acute radiation syndrome but also induces long-term (LT) bone marrow (BM) injury. The latter effect of IR is primarily attributed to the induction of hematopoietic stem cell (HSC) senescence. Granulocyte colony-stimulating factor (G-CSF) is the only treatment recommended to be given to radiation victims soon after IR. However, clinical studies have shown that G-CSF used to treat the leukopenia induced by radiotherapy or chemotherapy in patients can cause sustained low white blood cell counts in peripheral blood. It has been suggested that this adverse effect is caused by HSC and hematopoietic progenitor cell (HPC) proliferation and differentiation stimulated by G-CSF, which impairs HSC self-renewal and may exhaust the BM capacity to exacerbate IR-induced LT-BM injury.

**Methods:**

C57BL/6 mice were exposed to 4 Gy γ-rays of total body irradiation (TBI) at a dose-rate of 1.08 Gy per minute, and the mice were treated with G-CSF (1 μg/each by ip) or vehicle at 2 and 6 h after TBI on the first day and then twice every day for 6 days. All mice were killed one month after TBI for analysis of peripheral blood cell counts, bone marrow cellularity and long-term HSC (CD34-lineage-sca1+c-kit+) frequency. The colony-forming unit-granulocyte and macrophage (CFU-GM) ability of HPC was measured by colony-forming cell (CFC) assay, and the HSC self-renewal capacity was analyzed by BM transplantation. The levels of ROS production, the expression of phospho-p38 mitogen-activated protein kinase (p-p38) and p16^INK4a^ (p16) mRNA in HSCs were measured by flow cytometry and RT-PCR, respectively.

**Results:**

The results of our studies show that G-CSF administration mitigated TBI-induced decreases in WBC and the suppression of HPC function (CFU-GM) (p < 0.05), whereas G-CSF exacerbated the suppression of long-term HSC engraftment after transplantation one month after TBI (p < 0.05); The increase in HSC damage was associated with increased ROS production, activation of p38 mitogen-activated protein kinase (p38), induction of senescence in HSCs.

**Conclusion:**

Our findings suggest that although G-CSF administration can reduce ARS, it can also exacerbate TBI-induced LT-BM injury in part by promoting HSC senescence via the ROS-p38-p16 pathway.

## Background

The hematopoietic system is exceedingly sensitive to ionizing radiation (IR). Acute radiation syndromes (ARSs) such as infection, bleeding, anemia and other clinical manifestations are mainly because of acute bone marrow (BM) suppression induced by IR. BM suppression is a life-threatening hazard when exposure to a moderate to high dose of total body irradiation (TBI) [1, 2]. The hematopoietic progenitor cells (HPCs) and a small amount of hematopoietic stem cells (HSCs) undergo apoptosis after exposure to IR, thus resulting in acute BM suppression within days [3]. Its clinical manifestations could be successfully managed by the use of hematopoietic growth factors (HGFs) [4]. However, even though some irradiated patients recover from IR-induced acute myelosuppression, they may develop long-term BM injury manifested by decreasing the HSC reserves and damaging HSC self-renewal ability subsequently. Unlike acute bone marrow suppression, residual BM damage is larvaceous, and under homeostatic conditions, patients with residual BM damage usually have a prolonged period of normal blood cell counts despite decreasing in HSC reserves. However, the clinical manifestation of residual BM injury has been largely overlooked because of this latency. Moreover, the risk of residual BM damage has been failed to consider by the seemingly normal blood cell counts and BM cellularity, especially after HGFs treatment.

Granulocyte colony-stimulating factor (G-CSF) is an important member of the hematopoietic cytokine family secreted by immune and non-immune cells that can not only stimulate the proliferation and differentiation of hematopoietic cells [5] but also regulate the immune [6], nervous [7], and endocrine systems [8]; it also plays a major role as a regulator of hematopoiesis and innate immune responses [9, 10]. Stimulating the patient’s own marrow recovery through the use of G-CSF is therefore a potentially effective countermeasure in certain irradiated patients.

G-CSF can stimulate HPC proliferation and differentiation to reduce acute hematopoietic radiation injury [11, 12]. G-CSF can reduce the incidence of neutropenia associated with radiation- and chemotherapy-induced marrow aplasia [13–15], what’s more, G-CSF activate neutrophils to enhance it’s function, such as promoting microbiocidal activity, which is important for the host’s nonspecific immune response mediated by opportunistic infection [16, 17], and pegylated G-CSF (a longer half-life of G-CSF) has been reported to mitigate neutropenia, anemia, and thrombocytopenia in irradiated B6D2F1/J mice [18]. However, G-CSF treatment after chemotherapy may directly or indirectly promote hematopoietic stem cell (HSC) proliferation and differentiation, leading to HSC exhaustion [19, 20]; G-CSF used to treat the leukopenia induced by radiotherapy or chemotherapy in patients can cause sustained low white blood cells in peripheral blood. However, whether G-CSF accelerates HSC exhaustion after TBI and the associated mechanisms have yet to be elucidated [21]. The effects of G-CSF were therefore investigated using a sublethal dose TBI mouse model.

## Results

### G-CSF ameliorates ionizing radiation-induced reduction in peripheral blood cells

**Fig. 1:**
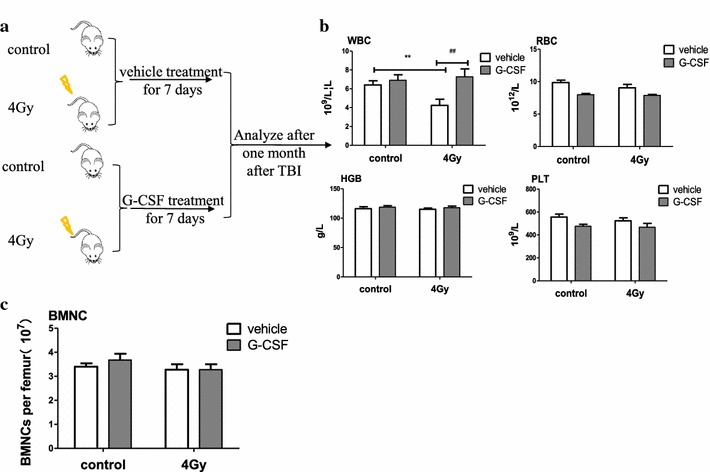
G-CSF treatment schedule. **a** Mice were divided into four groups randomly and treated daily with i.p. vehicle or G-CSF (1 µg/time, twice a day) for 7 days after exposure to 4 Gy TBI and then fed sterilely for 1 month after TBI. Sham-irradiated mice were treated in the same way regarded as a control. **b** The numbers of white blood cells (WBCs), red blood cells (RBCs), Hgb and platelets (PLTs) in peripheral blood were quantified after the mice were killed 1 month later. **c** Bone marrow nucleated (BMN) cells were quantified 1 month after various treatments. The data are presented as mean ± SEM, six mice/group. **p < 0.005 (vs. control); ^##^p < 0.005 (vs. 4 Gy)

Previous studies have showed that exposure to sub-lethal doses of total body γ-irradiation (TBI) not only leaded to acute BM injury but also resulted in long-term bone marrow (LT-BM) suppression [22–24]. To investigate how G-CSF treatment effects on IR-induced hemopoietic system injury, the numbers of peripheral blood cells and bone marrow mononuclear cells (BMMNCs) were analyzed 1 month after 4 Gy TBI. Mice irradiated with 4 Gy γ-rays received injections of rhG-CSF or saline according to the schedules indicated in Fig. 1a. Peripheral blood was obtained via the orbital sinus 1 month after 4 Gy TBI, and WBCs, platelets, and hematocrit were measured as described previously [25]. As shown in Fig. 1b, the number of WBCs in vehicle-treated mice showed an obvious reduction 1 month after 4 Gy TBI compared with the control mice, but there was no difference in number of RBCs, HBG, and PLT between control and TBI mice. G-CSF treatment alone had no effect on peripheral blood counts compared with control mice, whereas G-CSF administration attenuated the reduction in the number of WBCs in 4 Gy TBI group, and it almost recovered the number of WBCs after exposure to 4 Gy TBI. However, there was no significant difference in the number of BMMNCs among all groups (two separate studies), as shown in Fig. 1c, suggesting that bone marrow cells recovered to normal levels 1 month after 4 Gy TBI. This finding indicates that G-CSF can relieve IR-induced acute BM injury effectively.

### G-CSF did not affect the phenotype in hematopoietic cells dramatically

**Fig. 2:**
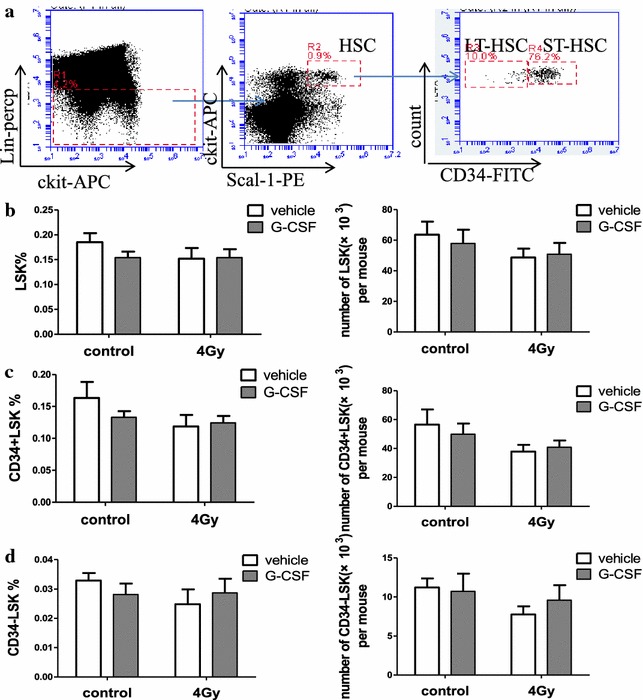
The effect of G-CSF on the frequency and number of long-term HSCs 1 month after TBI. Mice received vehicle or G-CSF treatment twice a day for 7 days after exposure to 0 or 4 Gy TBI as illustrated in Fig. 1a. The BM cells were collected after the mice were killed 1 month after various treatments and analyzed for long-term HSCs and short-term HSCs by flow cytometry. **a** Arepresentative gating strategy of LT-HSC and ST-HSC was analyzed by flow cytometry; **b** The frequencies of the LT-HSCs (CD34 + lineage-scal + ckit + cells) and the numbers of LT-HSCs pre mouse; **c** The frequencies of the ST-HSCs (CD34 + lineage-scal + ckit + cells) and the numbers of ST-HSCs pre mouse; **d** The frequencies of HSCs (lineage-scal + ckit + cells) and the numbers of HSCs per mouse. The data are presented as mean ± SEM, six mice/group

IR could reduce the number and frequency of HSPCs (hematopoietic stem and progenitor cells), and accelerate the HSC exhaustion. Our results show that BMNCs returned to normal levels 1 month after TBI (Fig. 1c). To investigate the effect of G-CSF on the frequency and numbers of LSK (lin-scal + ckit+), ST-HSCs (CD34 + lin-scal + ckit+) and LT-HSCs (CD34-lineage-scal + ckit+) after exposure to 4 Gy TBI, we analyzed the frequencies and numbers of different hematopoietic cell populations in BM cells by flow cytometry, as shown in Fig. 2a. The results of this assay revealed that there were no significant differences in the frequencies or numbers of LSK, ST-HSCs and LT-HSCs between irradiated and control mice 1 month after TBI (Fig. 2b, c, d). Further analysis revealed that G-CSF administration after 4 Gy TBI did not significantly change the frequencies or numbers of LSK, ST-HSCs, and LT-HSCs in BM cells (Fig. 2b, c, d). This result suggests that G-CSF did not affect the phenotype in different populations of hematopoietic cells one month after 4 GyTBI.

### G-CSF mitigated TBI-induced suppression of HPC function

**Fig. 3:**
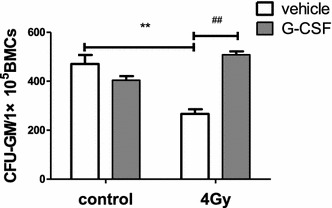
G-CSF administration reduced TBI-induced suppression of HPC proliferation. Mice received vehicle or G-CSF treatment twice a day for 7 days after exposure to 0 or 4 Gy TBI as illustrated in Fig. 1a. BMMNCs were harvest from the sacrificed mice 1 month after various treatments and cultured in MethoCult GF M3534 methylcellulose medium for 7 days, and then analyzed the colony-forming unit-granulocytes and macrophages (CFU-GM). The data are presented as mean ± SEM, six mice/group. **p < 0.005 vs. control; ^##^p < 0.005 (vs. 4 Gy)

In addition, we performed a CFC assay to examine whether hematopoietic cells from irradiated mice maintained their clonogenic function after G-CSF treatment, as illustrated in Fig. 3. As expected, radiation exposure significantly reduced the production of colony forming units-granulocyte–macrophage (CFU-GM) 1 month after 4 Gy TBI by 43.6 %. Whereas mice that received G-CSF treatment after exposure to 4 Gy TBI increased the production of CFU-GM dramatically, G-CSF treatment mice did not show a significant difference compared with the control mice. These results suggest that G-CSF could attenuate the radiation-induced suppression of hematopoietic progenitor cells.

### Effect of G-CSF on TBI-induced LT-BM injury

**Fig. 4:**
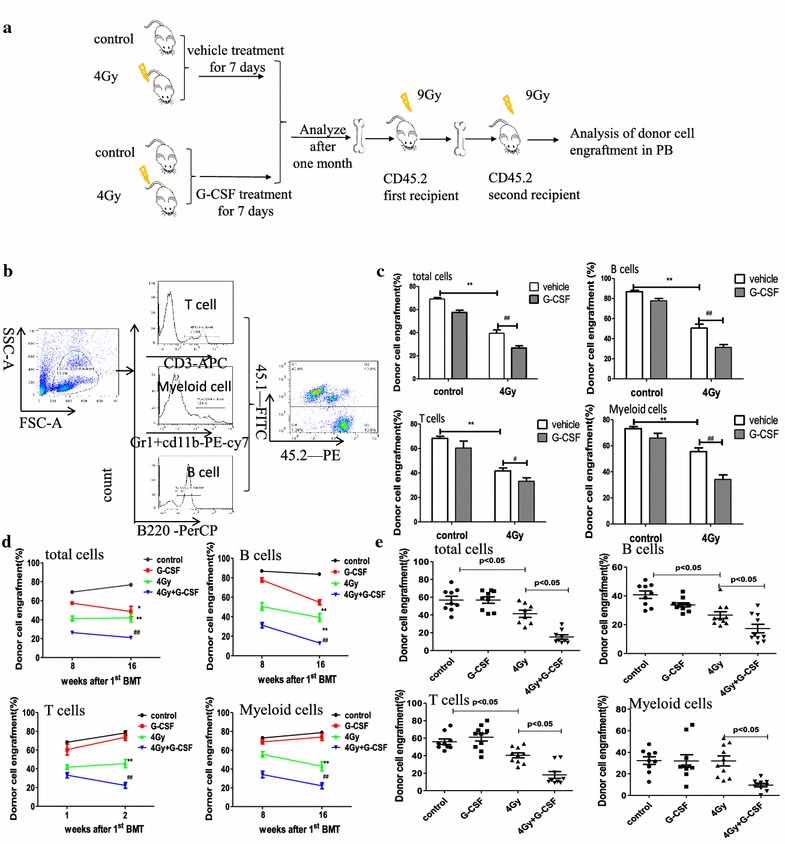
G-CSF exacerbated the HSCs long-term and multilineage engraftment reduction induced by TBI. **a** Competitive transplantation assay were performed by transplanting a 4:1 ratio of 45.1 donor cells to 45.2 competitive cells into 9.5 Gy-irradiated recipients. After stable hematopoietic reconstitution (16 weeks), mice were sacrificed, and bone marrow cells were harvested and transplanted into secondary recipients in the same way as the first competitive assay. A peripheral blood chimera for total cells, T cells, and B cells was assessed after 16 weeks. **b** Representative flow cytomerty plots showing donor-derived cell engraftment in peripheral blood cells. Right: percentage of donor (CD45.1+) cell engraftment. *Left*: engraftment of donor-derived T cells (CD45.1 + CD3 + cells), B cells (CD45.1 + B220 + cells), and myeloid cells (CD45.1 + CD11 + and/or Gr-1 + granulocyte–monocyte–macrophage) in the peripheral blood. **c** Donor -derived bone marrow cells multilineage engraftments were determined at 8 weeks in lethally irradiated recipients after first transplantation. **d** Donor -derived bone marrow cells multilineage engraftments tendency were measured at 8 and 16 weeks in lethally irradiated recipients after first transplantation. **e** Donor -derived bone marrow cells multilineage engraftments were determined at 16 weeks in lethally irradiated recipients after second transplantation of BM-MNCs from mice receiving various treatments described in Fig. 1a. Results are presented as mean ± SE. * p < 0.05, ** p < 0.005 vs. control; ^#^ p < 0.05, ^##^ p < 0.005 (vs. 4 Gy) (N = 9–12 recipient mice/group)

A previous study showed that G-CSF exacerbated hematopoietic stem cell injury caused by repeated administration of cytotoxic agents [20]. To investigate whether G-CSF treatment reinforced HSC long-term injury induced by IR, competitive repopulation assays were performed. We used the whole bone marrow from Ly5.1 mice after receiving vehicle or G-CSF treatment according to the schedule illustrated in Fig. 1a as donor cells. Donor cells with the competitive cells from Ly5.2 mice bone marrow were injected together into the Ly5.2 recipients in a 4:1 ratio. The donor derived T, B -lymphocytic and myeloid lineage engraftments in the peripheral blood cells of recipient mice were measured 8 and 16 weeks after 1^st^ transplantation (Fig. 4c, d), and 16 weeks after 2nd transplantation (Fig. 4e).

As expected, the untreated donor cells remained stable engraftment. However, the HSCs from vehicle treated mice after expose to 4 Gy TBI dramatically decreased the long-term and multilineage engraftment by approximately forty percent after first transplantation compared with that from control mice (Fig. 4b). These findings indicate that mice exposure to a sublethal dose of TBI (4.0 Gy) could cause long-term BM injury. Our results show that G-CSF treatment after exposure to 4 Gy TBI would exacerbate the LT-HSC injury compared with 4 Gy irradiated mice, which is manifestated by a further decreased donor-derived leukocytes, T cells, B cells, and myeloid cells engraftment in peripheral blood at 8 weeks and 16 weeks after the first transplantation (Fig. 4d). Intriguingly, G-CSF treatment alone 1 month later led to a decrease in the engraftment of donor-derived cells 16 weeks after first transplantation.

To assess the self-renewal capacity of HSCs, secondary bone marrow transplantations were performed. Donor derived multilineage engraftment was maintained from the vehicle-treated and G-CSF-treated mice in secondary recipients, 4 Gy irradiated donor mice bone marrow cells showed a marked decreased engraftment, and G-CSF treated mice after exposure to 4 Gy TBI would further exacerbate the injury, donor-derived leukocytes, T cells, B cells, and myeloid cells in the peripheral blood showed a further decreased engraftment 16 weeks after second transplantation (Fig. 4e). Thus, G-CSF further reduced the repopulating and self-renewal capacity of HSCs induced by IR.

### G-CSF promoted TBI-induced increases in ROS production in HSCs

**Fig. 5:**
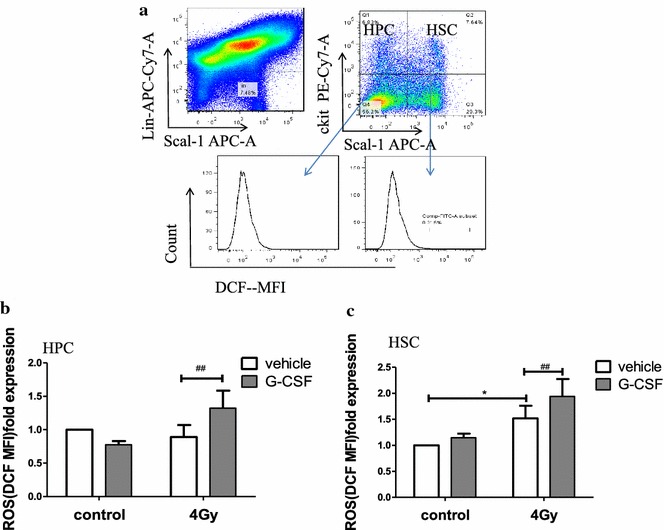
G-CSF treatment deteriorates the TBI-induced increase in ROS production in HSCs. Mice received vehicle or G-CSF treatment twice a day for 7 days after exposure to 0 or 4 Gy TBI as illustrated in Fig. 1a. BM-MNCs were harvested from scarified mice 1 month after exposure to TBI. The ROS production in HPCs and HSCs were analyzed. **a** Flow cytometric analysis was used to to estimate the ROS production in HPCs and HSCs. **b** The levels of intracellular ROS fold expression in HPCs are presented as the mean ± SE of DCF MFI. **c** The levels of intracellular ROS fold expression in HSCs are presented as the mean ± SE of DCF MFI

TBI could cause long-term BM suppression by inducing chronic oxidative stress and senescence in HSCs [26, 27]. Previous study showed that exposure to a sublethal dose of TBI induced oxidative stress reaction in HSCs selectively [28]. In our study, the production of ROS was dramatically increased in HSCs rather than in HPCs from vehicle-treated mice after exposure to 4 Gy TBI, as shown in Fig. 5b, indicating that persistent oxidative stress existed in HSCs 1 month after TBI. Intriguingly, G-CSF administration after exposure to 4 Gy TBI led to a much higher level of ROS in HSCs than that in the 4 Gy TBI group and G-CSF group. These findings suggest that G-CSF further exacerbates the chronic oxidative stress in HSCs induced by IR, thus deteriorating TBI-caused long-term BM suppression.

### G-CSF promotes TBI-induced increase in p38 expression and senescence in HSCs

**Fig. 6:**
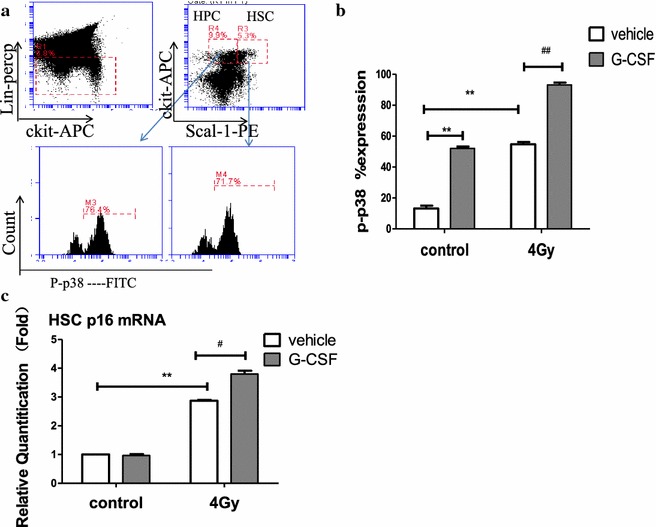
G-CSF treatment increased TBI-induced p38 expression and senescence in HSCs. Mice received vehicle or G-CSF treatment twice a day for 7 days after exposure to 0 or 4 Gy TBI as illustrated in Fig. 1a. The BM cells were collected after the mice were killed 1 month after receiving various treatments, and then p38 activation in HSCs (lineage-ckit + scal +, LKS +) was analyzed by flow cytometry. **a** Representative flow cytometric analysis of phosphorylated p38 (p38) in HSC cells, **b** the percentages of p-p38 positive cells under various treatment conditions are presented as mean ± SE (n = 5), **c** the levels of p16mRNA expression in sorted HSCs were analyzed by RT-PCR and are expressed as mean ± SE of fold changes compared to control (n = 3)

Ionizing radiation leads to the suppression of HSC function, partly through the induction of oxidative stress in HSCs, and p38 is of great importance to regulate the HSC self-renewal, and it can be activated by ROS. In addition, p38 involves in mediating radiation-induced HSC senescence, which is an element attributable to long-term bone marrow injury [29]. In our experiment, G-CSF exacerbated the HSC injury induced by IR, characterized by decreased repopulation and self-renewal functions. To determine whether G-CSF compromised HSC function by activating the ROS-p38-p16 pathway to promote the induction of HSC senescence, we measured the intracellular p38 phosphorylation (p-p38) and the p16 mRNA expression, which have been used as biomarkers of HSC senescence.As shown in Fig. 6b, 4Gy TBI resulted in a dramatic increase in p38 activation in HSCs (lineage-scal + ckit+ cells). G-CSF treatment alone also resulted in a high-level of expression of p-p38. G-CSF administration induced a much higher level of p-p38 in HSCs 1 month after TBI compared with that in TBI group. Moreover, our results showed that exposure to radiation significantly increased the expression of p16 mRNA in HSCs and G-CSF administration potentiated the induction (Fig. 6c). These results suggest that G-CSF promoted radiation-induced senescence in bone marrow hematopoietic cells, in part via the ROS-p38-p16 pathway.

## Discussion

G-CSF mobilizes HSCs to peripheral blood and increases peripheral leukocyte counts; thus, G-CSF is widely used in the treatment of myelosuppression induced by radiotherapy and chemotherapy. However, it has been shown in experimental models that, G-CSF administration may have some adverse effects on hematopoietic stem cells after receiving multiple doses of cytotoxic agents treatments, which themselves could induce the primitive stem cells injury [20]. Ronald et al. also reported that G-CSF impeded bone marrow cells recovery from damage caused by cytotoxic agents through increasing differentiation at the expense of self-renewal [19]. Schuettpelz et al. reported that Toll-like receptor (TLR) expression and signaling after G-CSF treatment resulted in phenotypic HSCs expansion but reduced their repopulation and self-renewal ability [30]. However, whether G-CSF exacerbates IR-induced HSC function and the mechanisms have yet to be elucidated. In our study, we found that TBI decreased WBC counts in PB and CFU-GM and induced persistent oxidative stress, intracellular phospho-p38 expression and senescence in HSCs. Treatment with G-CSF effectively inhibited TBI-induced reduction of WBC and HSC clonogenic function defects. Our experiments also showed that G-CSF could damage long-term HSC repopulating and self-renewal ability, which could further compromise long-term HSC function after IR. In our study, G-CSF administration could exacerbate TBI-induced long-term repopulation activity defects by further increasing ROS production and p38. A previous study showed that G-CSF combined with the p38 inhibitor SB203580 could efficiently increase the clonogenic function of BM hematopoietic progenitor cells (HPCs) and the frequency of HSCs, ST-HSCs and LT-HSCs 30 days after TBI, What’s more, the combination of G-CSF and SB203580 could increased the cobblestone area-forming cell (CAFC) of HSCs of irradiated mice [22, 31], which is an important indicator of HSC function, suggesting that G-CSF treatment may damage HSC function in part by inducing ROS-p38 oxidative signal expression. HSC senescence is an important factor contributing to long-term bone marrow suppression, TBI could induce HSC senescence, we found that G-CSF treatment after TBI would further aggravating HSC senescence, which was indicated by further increased p16 mRNA expression.

Wang et al. previously reported the p38 pathway plays an important role in mediating hematopoietic stem cell senescence induced by IR, and it can be activated by oxidative stress, and pharmacological inhibition of p38 can effectively abate IR-induced residual BM injury [32, 33]. The authors observed that p38 was selectively activated in irradiated hematopoietic cells, and the long-term BM cell culture assay revealed that the activation of p38 was sustained for up to 5 weeks after IR. Inhibition of p38 activity with a specific inhibitor, such as SB203580, abated IR-induced suppression of BM hematopoietic cell function along with a significant reduction in p16 expression and SA-b-gal activity. These findings and our results demonstrated that the activation of p38 by oxidative stress can serve as a prominent mediator to induce the LT-HSC bone marrow suppression. G-CSF plays an important role in activating p38 expression and HSC senescence.

In addition, G-CSF signaling results in the suppression of osteoblast lineage cells in monocytic cells, and the osteoblast lineage cells play an important role in maintaining HSPCs in the bone marrow [34]. Singh et al. demonstrated that neutrophil expansion, a physiological consequence response to G-CSF, could lead to the apoptosis in osteolineage cell populations of BM and reduce the production of HSC retention factors such as SDF-1, SCF, and VCAM-1, so it can disrupt the BM niche, and lead to HSPC mobilization [15]. G-CSF promotes HSC mobilization, likely affects the niche signaling, then reduces HSC maintaining in stroma, and exacerbates the exhaustion of the HSC pool; however, such assumptions still require further experimental validation.

Previous studies have not reported on how G-CSF administration affects the number and function of long-term hematopoietic stem cells after TBI. For the first time, our results show that G-CSF would not affect the number of LSK cells and HSCs but would further reduce the function of long-term hematopoietic stem cells 1 month after exposure to TBI, elevate the level of ROS, and induce the expression of p38; the results also suggest that G-CSF further impedes hematopoiesis in bone marrow injured by IR partly by increasing ROS-p38 oxidative signaling and HSC senescence. Our experiments also show that G-CSF administered alone compromised HSCs’ repopulating ability, particularly B cell engraftment after the first transplantation due to increased P38 activity, which is not regulated by ROS. Based on previous studies, G-CSF can regulate HSC function by toll-like receptor signaling [35, 36], and p38 is an important downstream activating protein of toll-like receptor signaling [37]. Therefore, we suggest that G-CSF treatment alone compromises HSC function through inflammatory signals.

Singh et al. reported that Genistein, which is used as an antineoplastic and antitumor agent, can be combined with G-CSF to protect HSCs against G-CSF-induced DNA damage [38]. Li et al. also reported that G-CSF in combination with p38 inhibitors can increase CAFC of the HSCs injured by ionizing radiation. We expect that a combination of such drugs with G-CSF can effectively mobilize HSCs, reducing the clinical symptoms of acute radiation injury and decreasing the likelihood of further long-term bone marrow suppression. Our results show that G-CSF can decrease HSC function by exacerbating the ROS-p38 oxidative stress pathway and HSC senescence, suggesting that this antioxidant and anti-senesence agent could be a promising choice for combination. In conclusion, our hematopoietic stem cell experiments provide a new perspective on the application of radiotherapy and treatment of acute radiation sickness, with the aim of reducing the side effects of clinical hematopoietic therapy based on stem cell mobilization.

## Methods

### Mice

Male C57BL/6-Ly-5.1 (Ly5.1) mice were purchased from the Institute of Laboratory Animal Sciences (PUMC, Beijing, China); C57BL/6 J (Ly5.2) mice were purchased from Vital River (Beijing, China).All the mice housed in the certified animal facility of the Institute of Radiation Medicine of the Chinese Academy of Medical Sciences (CAMS). Mice were 8–12 weeks of age, weighting 20–25 g. The Animal Use Committee at the Institute of Radiation Medicine of CAMS (No 1403) approved all the experimental procedures used during the research.

### TBI and G-CSF administration

Twenty four male mice were divided into four groups randomly: (a) control group; (b) 4 Gy group; (c) G-CSF group; (d) G-CSF + 4 Gy group. Mice from G-CSF + 4 Gy group and 4 Gy group were exposure to 4 Gy γ-rays (dose rate:1.08 Gy per minute) using an Exposure Instrument Cammacell-40 (Atomic Energy of Canada Lim), and then administered with G-CSF or vehicle (saline in 100 ul volume) twice a day for 7 days with intraperitoneal injections at an dose of 1 μg/mouse [25, 39]. The mice from G-CSF group and control group were not exposure to irradiation, and were treated in a manner similar to that described above.

### Peripheral blood cell and BM nucleated cell (BMNCs) counts

Mice were anesthetized, and then blood was obtained via the orbital sinus and was collected in anticoagulant tubes. The numbers of peripheral blood cells included white blood cells (WBCs), red blood cells, platelets, and the hemoglobin (HGB) were measured by an MEK-7222 K (Nihonkohden, Japan). BM cells were harvested as previously described [25]. The number of viable BMNCs was counted and expressed as ×10^7^/femur.

### Hematopoietic cells phenotypic analysis by flow cytometry

Briefly, 1 × 10^6^ hematopoietic cells harvested from mouse bone marrow were incubated with biotin-conjugated lineage antibodies (CD4, CD8, CD45R/B220, Gr-1, Mac-1 and Ter-119) and then stained with streptavidin-Percp to separate the lineage-positive cells. The cells were washed with PBS and incubated with anti-CD16/CD32 antibody to block Fc receptors. Finally, the frequencies of HPCs (Lin–Sca1–c-kit1 + cells), LSK cells (Lin–Sca1 + c-kit + cells), short-term HSCs (ST-HSCs, CD34 + LSK cells), and long-term HSCs (LT-HSCs, CD34–LSK cells) were analyzed with a flow cytometer (BD Accuri C6, San Jose, CA). The data were analyzed using BD Accuri C6 software.

### Colony-forming cells (CFC) assay

The CFC assay was implemented by culturing BM cells in MethoCult GF M3534 methylcellulose medium (StemCell Technologies, BC, Canada), and the colony-forming unit-granulocyte macrophage (CFU-GM) with more than 30 cells were scored as a colony under an inverted microscope according to the manufacturer’s instructions. The results were expressed as the numbers of CFU-GM per 10^5^ BMNCs.

### Competitive repopulation assay (CAR)

Competitive repopulation assays were performed as described previously [26, 27]. Specifically, for first transplantation, the cells (1 × 10^6^ BMCs) were harvested from the donor Ly5.1 mice and then mixed with 2 × 10^5^ competitive BMCs from Ly5.2 mice. The mixed cells were transplanted into lethally irradiated (9.5 Gy TBI) Ly5.2 recipient mice though lateral canthus vein injection. For second transplantation, CD45.2 cells (1 × 10^6^ BMCs) sorted from 1st transplantation recipient mice accompany with 2 × 10^5^ competitive BMCs from Ly5.2 mice were transplanted into lethally irradiated (9.5 Gy) Ly5.2 s recipient mice. To analyze the donor cells engraftment, peripheral blood was harvested at 8 and 16 weeks after first transplantation and 16 weeks after second transplantation. The red blood cells were lysed by 1 × RBC lysis (eBioscience Inc. San Diego, CA, USA), and the blood samples were stained with anti-CD45.1-FITC, anti-CD45.2-PE, anti-CD3-APC, anti-B220-PerCP, and anti-Gr-1 and CD11b-PE/Cy7 and were analyzed by BD FACS AriaIII.

### Flow cytometric analysis of intracellular ROS

1 × 10^6^ BM lineage-negative hematopoietic cells were stained with anti-Sca-1–APC and anti-c-kit–PE-Cy7 antibodies and then incubated with DCFDA (10 μM) for 30 min at 37 °C. To evaluate the productions of ROS in HPCs and HSCs, the mean fluorescence intensity (MFI) of 2′, 7′-dichlorofluorescein (DCF) was measured by using a flow cytometer as described previously [26, 27].

### Analysis of p38 activation by phosphor-specific flow cytometry

Approximately 1 × 10^6^ freshly isolated BMNCs were first incubated with specific antibodies to separate the lineage negative cells (CD4, CD8, CD11b, CD45R/B220, Ter-119, and Gr-1- biotin-conjugated) and then stained with streptavidin-Percp, Sca-1-PE, and c-kit-APC. The cells were then fixed and permeabilized with BD Cytofix/Cytoperm™ Fixation/Permeabilization Solution Kit (BD Pharmingen, San Diego, CA), and stained with FITC conjugated with anti-p-p38 antibody (BD Pharmingen,San Diego, CA) (1:50) for one hour at 4 °C according to the manufacturers’ instructions. After washing with PBS, cells were analyzed on a flow cytometer, and the data were analyzed using BD Accuri C6 software (BD Accuri C6, San Jose, CA).

### Isolation of HSCs (Lin– c-kit+ Sca-1+ cells)

These experiments were performed as we previously reported [28, 40].

### Quantitative real-time PCR

Total cellular RNA was extracted from about 20,000 sorted HSCs using TRIzol reagent (Invitrogen, Carlsbad, CA) following the manufacturer’s instructions. RNA yield and quality were determined by measuring absorbencies at 260 and 280 nm, respectively. First-strand cDNA was synthesized from total RNA by RevertAid First Stand cDNA Synthesis Kit (Thermo scientific) according to the manufacturer’s protocol. Quantitative real-time PCR was performed using TaqMan PCR primers and MasterMix (Applied Biosystems). Taqman MGB probes for the p16Ink4a (catalog number: Mm00494449_m1), and- the housekeeping gene Gapdh (catalog number: Mm99999915_g1) were obtained from Applied Biosystems (Foster City, CA). All samples were analyzed in triplicate using an ABI Prism 7500 Sequence Detection System (Applied Biosystems). The threshold cycle (CT) values for each reaction were determined and averaged using TaqMan SDS analysis software (Applied Biosystems). The changes in gene expression were calculated by the comparative CT method (fold changes = 2^−△△^CT) as described previously [41].

### Statistical analysis

The differences between treated and control groups were examined by unpaired Student’s t tests. Differences were considered significant at p < 0.05. All of these analyses were performed using GraphPad Prism from GraphPad Software (San Diego, CA, USA).

## Abbreviations

IR: ionizing radiation
LT: long-term
BM: bone marrow
HSC: hematopoietic stem cell
HPC: hematopoietic progenitor cell
G-CSF: granulocyte colony-stimulating factor
TBI: total body irradiation
CFU-GM: colony-forming unit-granulocyte and macrophage
CFC: colony-forming cell
ARS: acute radiation syndromes
HGFs: hematopoietic growth factors
TLR: Toll-like receptor
WBC: white blood cells
HGB: hemoglobin
BMNCs: BM nucleated cell
HBSS: Hank’s balanced salt solution
LT-HSCs: long-term hematopoietic stem cell
ST-HSCs: short-term hematopoietic stem cell
MFI: mean fluorescence intensity
CAR: competitive repopulation assay
CAFC: cobblestone area-forming cell
MFI: fluorescence intensity
